# Effects of a neuromuscular joint-protective exercise therapy program for treatment of wrist osteoarthritis: a randomized controlled trial

**DOI:** 10.1186/s12891-023-07157-4

**Published:** 2024-01-05

**Authors:** Sara L. Larsson, Elisabeth Ekstrand, Lars B. Dahlin, Anders Björkman, Elisabeth Brogren

**Affiliations:** 1 Department of Hand Surgery, Skåne University Hospital, Malmö, Sweden; 2 Department of Translational Medicine - Hand Surgery, Lund University, Skåne University Hospital, Jan Waldenströms Gata 5, 205 03 Malmö, Sweden; 3 Department of Biomedical and Clinical Sciences, Linköping University, Linköping, Sweden; 4 Department of Hand Surgery, Institute of Clinical Sciences, Sahlgrenska University Hospital, Sahlgrenska Academy, University of Gothenburg, Gothenburg, Sweden; 5 Department of Health Sciences, Lund University, Lund, Sweden

**Keywords:** Wrist osteoarthritis, SLAC, SNAC, Exercise therapy, Neuromuscular control, Self-management, Randomized controlled trial

## Abstract

**Background:**

Individuals with wrist osteoarthritis (OA) can suffer from pain, muscular weakness, and impaired motion of the wrist, which can reduce the quality of life. While there is strong evidence that all patients with OA should receive first-line treatment with education and exercises, this approach has not yet been proposed for individuals with wrist OA. Therefore, this trial aimed to evaluate the effectiveness of a first line neuromuscular joint-protective exercise therapy program compared to a training program with range of motion (ROM) exercises in patients with wrist OA.

**Methods:**

In this randomized controlled trial (RCT), 48 patients with symptomatic and radiographically confirmed wrist OA were randomly allocated to a 12-week self-management program with either a neuromuscular joint-protective exercise therapy program (intervention group) or a training program with ROM exercises only (control group). Our primary outcome measure was the Patient-Rated Wrist Evaluation (PRWE) with secondary outcome measures of grip strength, range of wrist motion, the Numerical Pain Rating, Scale (NPRS), the Disabilities of the Arm, Shoulder, and Hand (DASH) and the Generalized Self-Efficacy Scale (GSES). The outcome measures were evaluated by a blinded assessor at baseline and 12 weeks. Between-groups differences were analyzed using the Mann–Whitney U test and within-group differences were analyzed with the Wilcoxon signed-rank test.

**Results:**

A total of 41 participants were analyzed at 12 weeks. There were no significant differences in PRWE between the groups at 12 weeks (*p* = 0.27). However, DASH improved significantly in the intervention group compared to the control group (*p* = 0.02) and NPRS on load within the intervention group (*p* = 0.006). The difference in DASH should be interpreted with caution since it could be due to a non-significant increase (worsening) from baseline in the control group in combination with a non-significant decrease (improvement) in the intervention group.

**Conclusions:**

This RCT showed that the novel neuromuscular joint-protective exercise therapy program was not superior in reducing pain and improving function compared to a training program with ROM exercises at 12 weeks. Future research is warranted to evaluate the effectiveness of forthcoming exercise therapy treatment programs for patients with wrist OA.

**Trial registration:**

ClinicalTrials.gov, NCT05367817. Retrospectively registered on 10/05/2022. https://clinicaltrials.gov.

## Background

The complex wrist joint is the fundament of the hand that enables us to grasp objects and move them with both force and precision [1]. Because of the role the wrist plays in daily activities, the joint is particularly at risk of injury and degenerative diseases, such as osteoarthritis (OA). In contrast to OA affecting the hand, knee and hip, wrist OA can occur at an earlier age and is more common in men [2]. Prior trauma to the wrist, such as fractures, dislocations, and ligament injuries, is the most prevalent cause of degenerative changes [3]. Two common types of wrist OA patterns are the scapholunate advanced collapse (SLAC), which is induced by a traumatic or degenerative scapholunate ligament injury, and the scaphoid non-union advanced collapse (SNAC), which is instigated by a non-union scaphoid fracture [4, 5]. The SLAC and SNAC leads to carpal instability, altered wrist kinematics and joint loading, with progressive arthritic degeneration of the radiocarpal and midcarpal joints [3].

Although individuals with wrist OA can have diverse experiences, pain is often the most central problem negatively affecting all aspects of life [6]. Living with wrist OA can be well tolerated for many years and in many cases, the individuals cannot recall a prior trauma even though radiographs show a SLAC or SNAC. However, the combination of development of chronic pain, limitations in wrist motion, and its impact on the level of activity and participation, makes most individuals with wrist OA seek medical care sooner or later [6].

Current treatment norms for wrist OA include alleviating pain and decreasing disability by splinting, non-steroidal anti-inflammatory medications, intraarticular steroid injections, and different surgical interventions [3]. However, for knee and hip OA, there is evidence that self-management programs, with education and exercises, can enable patients to manage symptoms, optimize their quality of life, and avoid or delay surgery [7–11]. For hand OA, in the thumb carpometacarpal (CMC) joint and the finger joints, the evidence of the effectiveness of education and exercises is weaker owing to the lack of high-quality studies [12, 13]. No previous studies have evaluated the effect a self-managed exercise therapy program can have on patients with wrist OA.

Regarding wrist rehabilitation in general, the theoretical importance of including neuromuscular exercises in the rehabilitation of the wrist has been highlighted in a few reviews [14–17]. Also, a small number of single case reports [18, 19], and cohort studies [20–22] indicate clinical benefits of exercise therapy programs following wrist ligament injuries, midcarpal instabilities, and chronic wrist pain. However, wrist exercise therapy programs have so far never been compared to other treatments or placebo, and thus the true effect is unknown.

This randomized controlled trial (RCT) aimed to investigate the effectiveness of a self-managed neuromuscular joint-protective exercise therapy program, described in a previous study protocol [23], compared to a training program with range of motion (ROM) exercises in patients with wrist OA. We evaluated these programs at 12 weeks with the Patient Rated Wrist Evaluation (PRWE) as our primary outcome [24]. Our hypothesis was that 12 weeks of self-managed exercise therapy would relieve pain and improve function more than the ROM training program.

## Material and methods

### Trial design

**Fig. 1:**
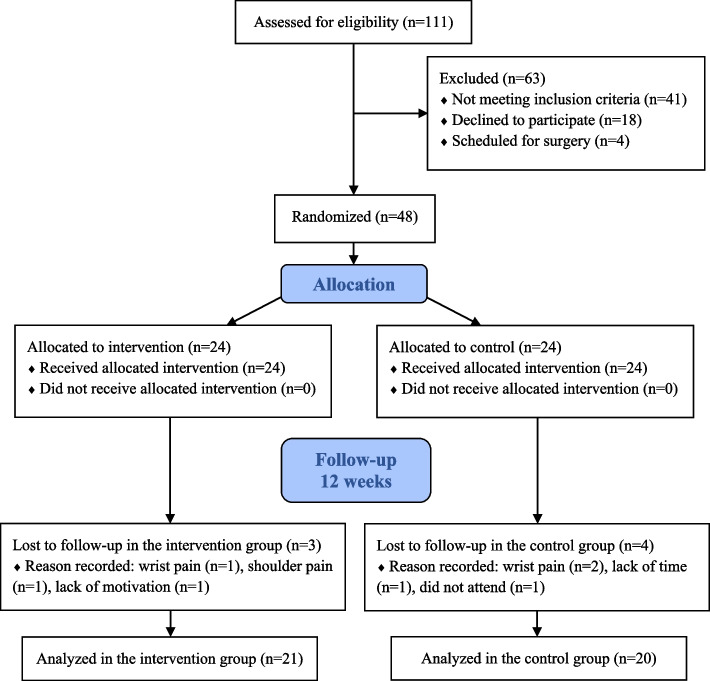
The Consolidated Standards of Reporting Trials (CONSORT) flow diagram of trial enrollment and follow-up

A randomized (1:1 ratio) controlled singled-blinded superiority trial with two treatment arms, was designed according to the Consolidated Standards of Reporting Trials (CONSORT) guidelines [25]. As outlined in the previously published study protocol [23], eligible participants were randomly allocated to an intervention group (exercise therapy program) or a control group (ROM training program) (Fig. 1). The trial was retrospectively registered at ClinicalTrials.gov on 10/05/2022 (identification number NCT05367817).

### Ethics

The Swedish Ethical Review Authority approved the trial, Dnr 2019–02437. The principles of the Declaration of Helsinki were followed. Prior to inclusion, information about the trial was provided and the participants gave their written informed consent to participate.

### Participants and setting

Participants were recruited at the Department of Hand Surgery, Skåne University Hospital, Malmö, Sweden. This is the main tertiary health care facility, where individuals with wrist OA are referred to, in the Southern health care region of Sweden with approximately 1.9 million inhabitants. Inclusion criteria were 1) radiographically confirmed and symptomatic wrist OA – SLAC and SNAC stage 1–3 [4], and 2) age ≥ 18 years. Exclusion criteria were 1) presence of other diseases or disorders that could affect arm and hand function, 2) wrist osteoarthritis secondary to avascular necrosis of carpal bones, 3) previous surgery to the wrist, 4) intraarticular wrist cortisone injection in the wrist within the last 3 months, and 5) inability to understand and follow test instructions due to communicative, mental, or cognitive impairments.

Individuals with symptomatic OA secondary to SLAC or SNAC, seeking care at the hand surgery clinic, were examined with conventional wrist radiographs in posterior-anterior and lateral views. Radiological diagnosis, in combination with clinical examination by the treating hand surgeon, confirmed the diagnosis of wrist OA. Potential participants were referred by the hand surgeon to the treating physiotherapist (PT) – the first author (SL) who is a specialist PT in treating hand and wrist injuries – who ensured that the inclusion criteria were fulfilled. Participants were then provided with all the relevant information about the trial and asked about participation. Included participants were also examined with computer tomography (CT) of the affected wrist to enable a detailed view of osteoarthritic signs. Two experienced specialist hand surgeons independently evaluated both the conventional radiographs and CT scans of the affected wrist and classified SLAC according to Watson and Ballet [4] and SNAC according to Vender et al. [5].

### Baseline assessment

Background information regarding 1) medical and social history, 2) demographic data, and 3) the use of pain medication was collected at the baseline assessment. The participants also reported, in pre-defined box alternatives, their main problem with the wrist, their main expectation of the allocated treatment program, if they had discussed surgical treatment with their hand surgeon and their own thoughts about surgery.

### Interventions

#### Trial treatments

A detailed explanation about the self-management strategies, the structured education, and the two treatment programs is reported in the study protocol [23].

In brief, participants were randomly assigned to undergo a 12-week self-management program, including structured education, and either a neuromuscular joint-protective exercise therapy program (intervention group) or a training program with ROM exercises (control group). Both groups received a booklet with information about wrist OA pathophysiology, the rationale behind exercise treatment, self-management strategies and activity modification principles. The participants were also instructed to apply the functional and most stable neutral wrist position in activities of daily living and were equipped with a stable wrist orthosis to wear, particularly during pain-provoking activities, but also at night-time if needed.

#### Procedure and adherence

The participants were instructed on how to perform the allocated exercise therapy program (intervention group) or the ROM training program (control group) on the same day as their baseline assessment. The exercises were instructed to be executed with good quality of movement—smooth, coordinated, and without compensatory movements—and the treatments were then continued as structured home-based programs that the participants performed twice a day for 12 weeks. The importance of adhering to the treatment programs was emphasized and the participants were followed up at the clinic by the treating PT at 2, 6, and 12 weeks after baseline and contacted by phone 4 and 8 weeks after baseline.

During the trial, they were able to take their usual pain medication, such as paracetamol or non-steroidal anti-inflammatory drugs (NSAIDs), if needed. However, pain medication, such as opioids, or intraarticular cortisone injection in the wrist were prohibited. A follow-up appointment with their treating hand surgeon was offered 3–6 months after inclusion.

#### Intervention group

The exercise therapy program contained structured education, a wrist orthosis, and exercises consisting of 1) active ROM exercises for the wrist in flexion/extension, radial-/ulnar deviation, and pronation/supination, and 2) neuromuscular exercises with focus on coordination, wrist stability, and strength. A detailed description of the program can be found in the study protocol [23]. The focus of the exercise therapy program was on functional re-learning and strengthening of the musculoskeletal system. The intention of the program was to create a stable wrist that could be used in a pain-free manner in daily activities.

#### Control group

The training program in the control group consisted of the same self-management strategies as in the intervention group with structured education and a wrist orthosis, but only included the above-mentioned ROM exercises.

### Outcome measures

Outcome measures with good psychometric properties was used covering both physical and patient-reported measures. Outcomes was assessed at baseline and 12-weeks post-inclusion. The Global Rating of Change (GROC) was not included in the 12-week follow-up, as stated in the study protocol [23], due to a late inclusion of the outcome measure. GROC will be analyzed in the future 6- and 12-month follow-ups.

#### Primary outcome

Pain and function was rated with the Swedish version of PRWE [26], a wrist specific outcome measure with strong psychometric properties in wrist OA [27]. The total score of PRWE is 100, which represents worst disability, whereas 0 represents no disability.

#### Secondary outcomes

Isometric grip strength, three trials for each hand with a mean value (kilograms, kg), was measured using the Jamar hydraulic hand dynamometer (TEC, Clifton, New Jersey, US) [28].

Range of wrist motion (flexion, extension, radial deviation, ulnar deviation, pronation, and supination) of the affected wrist was measured in degrees (°) with a goniometer [29].

Three aspects of pain – at rest, on motion without load, and on load – were rated with the numerical pain rating scale (NPRS), ranging from 0 (no pain) to 10 (worst pain imaginable) [30]. The NPRS have been found to be valid and reliable when measuring pain outcome in patients with wrist OA [27].

Self-reported upper extremity physical function and symptoms were rated with the Swedish version of the Disabilities of the Arm, Shoulder, and Hand (DASH) [31]. The total DASH score is 100 that represents most severe disability, whereas 0 characterise no disability. Strong psychometric properties have been found for DASH in patients with wrist OA [27].

Self-efficacy, the strength of a person’s belief in his or her ability to respond to a novel or difficult situation, was rated with the Generalized Self-Efficacy Scale GSES (Swedish version) [32]. The GSES score ranges from 10–40, where higher scores indicate greater generalized self-efficacy.

### Sample size

The sample size was calculated based on the minimal clinically important difference (MCID) of 12.5 for the primary outcome PRWE [33, 34]. With a standard deviation (SD) of 14, power (beta) at 0.8, a significance level (alpha) at 0.05, and a 2-tailed test, the power calculations indicated a sample size of 40 patients, 20 in each group. Accounting for a drop-out rate of 20%, a total of 48 patients were calculated to be included in the trial.

### Randomization

#### Sequence generation

Participants were randomly assigned to the intervention group or the control group by selecting a sealed envelope indicating the group allocation. The sequence was generated using block randomization with the size of 10 in each block.

#### Implementation

An occupational therapist (OT) at the hand surgery clinic, with no involvement in the clinical care of participants, generated the block randomization sequence. The envelopes were sealed and only opened by the treating PT on the same day as the baseline assessment when the participants were allocated either to the exercise therapy program (intervention group) or to the ROM training program (control group).

#### Blinding

Participants were not informed about which group they had been allocated to. An experienced blinded PT at the clinic performed all the evaluations at baseline and the 12-week follow-up. Also, the treating hand surgeons and the hand surgeons assessing the radiological wrist OA stage were blinded to group allocation. No adverse events or safety concern during the treatment period led to emergency unblinding.

### Statistical methods

Data were analyzed with IBM SPSS Statistics version 29 (IBM Corporation, Armonk, NY, USA). The intention-to-treat principle was used, but all participants were treated according to the group they were allocated to. As the data were not normally distributed according to the Shapiro-Wilks test, non-parametric tests were used in the analyses. Differences between the groups were analyzed using the Mann–Whitney U test and within-group differences were analyzed using the Wilcoxon signed-rank test. We used the Chi-square test to compare proportions in baseline characteristics between the groups. The level of statistical significance was set at *p* < 0.05.

## Results

### Participant flow

One-hundred and eleven patients were assessed for eligibility (Fig. 1). Forty-one did not meet the inclusion criteria. Eighteen patients declined to participate and four were already scheduled for surgery. These 22 non-participants did not differ in the distribution of sex (*p* = 0.30) but were, however, slightly older (*p* = 0.035) compared to the included participants. A total of 48 participants were included in the trial after informed consent and randomization allocated 24 in each group. Three participants in the intervention group and four participants in the control group dropped-out before completing the 12-week treatment (Fig. 1). Twenty-one participants in the intervention group and 20 in the control group were analyzed at 12 weeks (Fig. 1). No observable adverse events were noted at the 12-week follow-up and no participants reported any harm or discomfort. Trial recruitment started in October 2019 and was closed when we reached our sample size of 48 participants in March 2023.

### Baseline characteristics

There were no differences between the two groups in characteristics, patient-reported wrist function, pain, grip strength or range of motion at baseline (Tables 1 and 2). In both groups, the participants considered that pain was the main problem, and pain reduction was the main expectation with the allocated exercise program (Table 3). The median age in both groups was just above 60 years and most participants had wrist OA on their dominant side. There were more men included in both the intervention (83%) and the control (83%) groups. SLAC wrist was the most common cause of OA in both groups. A higher proportion of participants with SNAC wrist was seen in the intervention group (21%) compared to the control group (4%). A majority in both groups had grades 2–3 SLAC/SNAC. Inclusion of trial participants was done based on conventional radiographs and clinical symptoms. However, CT scans were also done but not analyzed until after inclusion. This resulted in the re-grading of five participants (two in the intervention group and three in the control group) from SLAC/SNAC 3 to 4.

**Table 1:** Comparison of baseline characteristics between the intervention group (exercise therapy program) and the control group (training program) in participants with wrist osteoarthritis

Variable	Intervention group (*n* = 24)	Control group (*n* = 24)	*p*-value
**Age**, median [IQR]	63 [55–69]	66 [56–70]	0.45
**Sex**, male, n (%)	20 (83)	20 (83)	0.65
**Occupation**, n (%)			
Retired/unemployed	8 (33)	11 (45.8)	0.19
Office-based duties	6 (25)	1 (4.2)	
Moderately heavy	8 (33)	8 (33.3)	
Manual labour	2 (8)	4 (16.7)	
**Type of OA**, n (%)			
SLAC	19 (79)	23 (96)	0.08
SNAC	5 (21)	1 (4)	
**OA grade**, n (%)			
Grade 1	0 (0)	3 (12.5)	0.30
Grade 2	10 (42)	9 (37.5)	
Grade 3	12 (50)	9 (37.5)	
Grade 4	2 (8)	3 (12.5)	
**Affected wrist**, **dominant**, n (%)	15 (63)	20 (83)	0.10
**Duration of symptoms**, n (%)			
0–2 years	9 (38)	11 (45.5)	0.75
3–6 years	7 (29)	4 (17)	
7–10 years	6 (25)	6 (25)	
> 10 years	2 (8)	3 (12.5)	
**Use of pain medication**, yes, n (%)	14 (58)	15 (63)	0.77

Values are numbers (n), medians, interquartile range [IQR] and percentages (%)

*OA* Osteoarthritis, *SLAC* Scapholunate Advanced Collapse, *SNAC* Scaphoid Non-union Advanced Collapse

**Table 2:** Comparison of baseline characteristics of the primary and secondary outcomes between the intervention group (exercise therapy program) and the control group (training program) in participants with wrist osteoarthritis

**Outcome measure**	**Intervention group (*n* = 24)**	**Control group (*n* = 24)**	***p*-value**
**PRWE**			
Pain	31 [19–39]	31 [22–35]	0.81
Function	20 [12–25]	25 [16–32]	0.17
Total	51 [33–67]	56 [40–64]	0.54
**DASH**	31 [21–41]	36 [28–50]	0.29
**NPRS**			
At rest	3 [1–5]	3 [1–5]	0.86
On motion without load	7 [4–8]	5 [3–8]	0.37
On load	8 [7–9]	8 [5–8]	0.07
**GSES**	32 [28–36]	31 [27–35]	0.73
**Wrist ROM (°)**^a^			
Extension	43 [30–55]	50 [36–55]	0.63
Flexion	40 [23–45]	30 [20–40]	0.18
Radialdeviation	10 [5–10]	10 [5–10]	0.78
Ulnardeviation	20 [15–25]	20 [20–30]	0.34
Pronation	70 [60–70]	70 [61–74]	0.56
Supination	78 [66–80]	75 [65–80]	0.43
**Grip strength**^a^	28 [23–36]	26 [18–37]	0.70

Values are medians and interquartile range [IQR], if not specified as degrees (°)

*PRWE* Patient-Rated Wrist Evaluation, *DASH* Disabilities of the Arm, Shoulder, and Hand, *NPRS* Numerical Pain Rating Scale, *GSES* Generalized Self-Efficacy Scale, *ROM* Range of Motion

^a^Wrist ROM and grip strength were measured on the affected wrist and hand

**Table 3:** Baseline characteristics of the predefined box-alternative questions in the intervention group (exercise therapy program) and the control group (training program) in participants with wrist osteoarthritis

Pre-defined questions	Intervention group (*n* = 23)^a^	Control group (*n* = 24)
**Main problem with the wrist, n (%)**		
Pain	22 (96)	21 (88)
Stiffness	1 (4)	0 (0)
Impaired grip strength	0 (0)	3 (12)
**Main expectation of exercise treatment, n (%)**		
Reduced pain	20 (87)	19 (79)
Improved range of wrist motion	1 (4.3)	0 (0)
Improved strength	1 (4.3)	4 (17)
Don’t know	1 (4.3)	1 (4)
**Discussed surgery, n (%)**		
Yes	21 (91)	19 (79)
No	2 (9)	5 (21)
**Views on surgery, n (%)**^b^		
Yes	3 (13)	4 (17)
No	12 (52)	7 (29)
If pain gets worse	6 (26)	2 (8)
Avoid as long as possible	2 (9)	7 (29)
Don’t know	0 (0)	3 (13)

Values are numbers (n) and percentages (%)

^a^One missing in the intervention group

^b^One missing in the control group

### Primary outcome

There were no statistically significant differences in our primary outcome PRWE between the groups at 12 weeks, neither for the subscales nor the total sum score (Table 4). There were also no significant within-group differences from baseline to 12 weeks.

**Table 4:** Between-group comparisons of the primary and secondary outcomes at 12 weeks between the intervention group (exercise therapy program) and the control group (training program) in participants with wrist osteoarthritis

Outcome measure	Intervention group (*n* = 21)	Control group (*n* = 20)	*p*-value
**PRWE**			
Pain	27 [13–34]	28 [21–36]	0.82
Function	16 [5–28]	25 [19–33]	0.13
Total	46 [16–63]	52 [41–68]	0.27
**DASH**	24 [13–35]	43 [26–53]	**0.02**
**NPRS**			
At rest	3 [0–5]	4 [2–7]	0.12
On motion without load	4 [2–7]	5 [3–8]	0.25
On load	6 [4–8]	7 [5–8]	0.69
**GSES**	33 [24–37]	32 [28–36]	0.76
**Wrist ROM (°)**^ab^			
Extension	48 [36–60]	45 [35–54]	0.53
Flexion	45 [30–45]	30 [20–50]	0.25
Radialdeviation	10 [6–15]	7.5 [5–10]	0.09
Ulnardeviation	20 [20–30]	20 [20–25]	0.95
Pronation	70 [61–75]	70 [65–79]	0.82
Supination	78 [70–80]	72 [70–80]	0.34
**Grip strength**^ab^	28 [22–39]	29 [22–34]	0.95

Values are medians and interquartile range [IQR], if not specified as degrees (°)

*PRWE* Patient Rated Wrist Evaluation, *DASH* Disabilities of the Arm, Shoulder, and Hand, *NPRS* Numerical Pain Rating Scale, *GSES* Generalized Self-Efficacy Scale, *ROM* Range Of Motion

^a^Missing values in control group; 1 participant

^b^Wrist ROM and grip strength were measured on the affected wrist and hand

### Secondary outcomes

#### Between-group differences

A significant difference in DASH score was seen between the groups at 12 weeks, with better patient-reported hand function in the intervention group (*p* = 0.02) (Table 4). For the other secondary outcomes, no significant differences between the groups were found (Table 4).

#### Within-group differences

From baseline to 12 weeks, some statistically significant differences were seen for wrist ROM (flexion and radialdeviation) and NPRS on load in the intervention group (Table 5). The reduction in median NPRS on load (2 points) was also a clinical important difference [35]. For the control group statistically significant, but not clinically important, differences were found for GSES and grip strength (Table 5).

**Table 5:** Within-group comparisons of the primary and secondary outcomes within the intervention group (exercise therapy program) and the control group (training program) in participants with wrist osteoarthritis

Outcome	Intervention group (*n* = 21)			Control group (*n* = 20)		
	**Baseline**	**12 weeks**	***p*-value**	**Baseline**	**12 weeks**	***p*-value**
**PRWE**						
Pain	31 [20–39]	27 [13–34]	0.13	31 [23–37]	28 [21–36]	0.13
Function	20 [11–24]	16 [5–28]	0.17	25 [19–35]	25 [19–33]	0.38
Total	50 [34–60]	46 [16–63]	0.13	56 [44–68]	52 [41–68]	0.17
**DASH**	31 [21–40]	24 [13–35]	0.09	36 [29–52]	43 [26–53]	0.91
**NPRS**						
At rest	3 [1–5]	3 [0–5]	0.44	3 [2–5]	4 [2–7]	0.46
On motion without load	6 [4–8]	4 [2–7]	0.07	5 [3–8]	5 [3–8]	0.61
On load	8 [7–9]	6 [4–8]	**0.006**	8 [4–8]	7 [5–8]	0.31
**GSES**	32 [28–36]	33 [24–37]	0.55	31 (27–35)	32 [28–36]	**0.04**
**Wrist ROM (°)**^ab^						
Extension	45 [30–55]	48 [36–60]	0.50	50 [35–55]	45 [35–54]	0.50
Flexion	40 [25–48]	45 [30–45]	**0.03**	30 [20–40]	30 [20–50]	0.12
Radialdeviation	10 [5–10]	10 [6–15]	**0.03**	5 [1–10]	7.5 [5–10]	0.78
Ulnardeviation	20 [18–25]	20 [20–30]	0.52	20 [16–25]	20 [20–25]	0.22
Pronation	70 [60–73]	70 [61–75]	0.23	70 [61–74]	70 [65–79]	0.41
Supination	75 [68–80]	78 [70–80]	0.33	75 [65–80]	72 [70–80]	0.85
**Grip strength**^ab^	29 [24–36]	28 [22–39]	0.06	26 [18–37]	29 [22–34]	**0.02**

Values are medians and interquartile range [IQR], if not specified as degrees (**°**)

*PRWE* Patient Rated Wrist Evaluation, *DASH* Disabilities of the Arm, Shoulder, and Hand, *NPRS* Numerical Pain Rating Scale, *GSES* Generalized Self-Efficacy Scale, *ROM* Range Of Motion

^a^Missing values in control group; 1 participant

^b^Wrist ROM and grip strength were measured on the affected wrist and hand

## Discussion

This prospective RCT, evaluating a novel self-managed neuromuscular joint-protective exercise therapy program for patients with wrist OA, showed that such a program was not superior in reducing pain and improving function compared to a ROM training program at 12 weeks.

Regarding our primary outcome PRWE, there was no significant difference neither between the groups nor within the groups at 12 weeks. For our secondary outcome measures, there were no overall significant differences, with a few exceptions. The intervention group had a significantly lower DASH score compared to the control group and a significant within-group decrease of NPRS on load at 12 weeks. The difference in DASH score should be interpreted with caution since it could be due to a non-significant increase (worsening) from baseline in the control group in combination with a non-significant decrease (improvement) in the intervention group. However, the significant and clinically important decrease in pain on load within the intervention group could be due to the joint-protective and neuromuscular nature of the exercise therapy program. Overuse of a joint affected by OA may increase the inflammatory process and synovitis leading to pain and fatigue [36]. These symptoms induce less efficient ways of using the joint in daily activities and an increase in unhealthy loading, which in turn can enhance the symptoms [37]. Joint-protective programs emphasize a better spread of the load across multiple joints to reduce the unhealthy loading of the OA affected joint [37]. This, in combination with strengthening specific muscle support, using energy-conserving techniques and adaptive equipment, could all reduce the stress on the joint which could explain the decreased pain on load within the intervention group [37].

Compared to hand and wrist OA, the evidence and recommendations regarding self-management programs and exercises are much stronger for knee and hip OA [9, 10, 38]. The effects of exercise in people with knee OA have been evaluated in more than 50 RCTs [7], and for people with hip OA in around 10 RCTs [8]. To summarize, these studies showed that pain and function improved following exercises in people with knee and/or hip OA. Even though positive results, such as decreased pain, improved function and improved precision on force sense, have been reported in recent studies for exercise training in patients with CMC OA, the effects in these studies are more limited [39–41]. The underlying mechanisms behind the more positive effects following exercises in people with knee and/or hip OA are, however, inadequately understood [42]. One difference could be a general positive physiological response, including weight loss, to the cardiovascular exercise training usually incorporated when treating knee and hip OA [43]. Our exercise therapy program has focused on increasing the muscle strength around the specific OA affected joint. However, this may not be sufficient enough to reduce pain and enhance function. Since joint protection aims at distributing the load over several joints and using the strongest, largest joint available for the task, we may need to incorporate the more proximal part of the upper extremity when designing future exercise programs for wrist and hand OA.

Patients with knee, hip and hand OA are most often first-line treated in primary care settings [44, 45]. In this sense, the exercise therapy program used in our trial differs since it took place in a tertiary care setting where patients are admitted with more advanced stages of OA and often with more severe symptoms. Early referral of patients with wrist OA for treatment with neuromuscular joint-protective exercises may improve outcomes to a greater extent than referral at a later stage [46]. Hence, late referral could be a factor that have affected the outcomes of our trial. However, patients with knee OA benefit from supervised exercise therapy regardless of grade of OA severity, which implies that all OA patients should be offered a first-line treatment approach with exercise therapy [47]. Perhaps, if patients with wrist OA would be more acknowledged in primary care and included in self-management treatment programs early before the SLAC and SNAC progression occurs, the benefits of such programs could be greater.

Our exercise therapy program was standardized in all parts, including the structured education, ergonomic advice, wrist orthosis and the neuromuscular exercises. However, this standardized approach may not be beneficial for all wrist OA patients, which could have affected the exercise therapy programs’ effect on pain and function in our trial. Some participants might have benefited from a more individualized program. This raises the question of the most optimal type of exercise and setting for an exercise therapy program for patients with wrist OA. A supervised and individualized exercise therapy treatment with a frequency of two supervised sessions per week in a total of 12 weeks is most recommended when treating knee and hip OA [48]. This stands in contrast to our exercise therapy program, and other exercise treatment programs for hand OA, that usually consist of home-based exercises with regular follow-ups at the clinic [39, 40]. Maybe, when treating wrist OA patients, this should be done in a supervised setting at the clinic since it could enhance the individualization of the program.

How well patients adhere to the program is of particular importance in a self-managed home exercise program. While some OA patients do well with an exercise home program, others struggle more and fail to do as well [49]. To promote adherence in our trial, both treatment programs were delivered and supervised by the experienced treating PT, who encouraged, answered questions, and ensured a correct performance of the exercises. Follow-ups were scheduled every second week during the exercise period, which has been found to support patient adherence [50]. Therefore, the level of adherence to the treatment programs in both groups should not have affected the results of this trial. However, digital applications with reminders or other adherence techniques could potentially further improve adherence of a self-managed exercise therapy program [51].

### Strengths and limitations

Even though we reached our power-calculated sample size, 41 participants might not be sufficient to detect potential differences between the two groups. The 12 weeks follow-up time frame can be seen as a limitation, however, future 6- and 12-month follow-ups are planned for these participants, as outlined in the study protocol [23]. Also, the exercise therapy program can be seen as more challenging for the participants in the intervention group due to the larger number of exercises, which could have affected their adherence to the program. We did not collect data regarding adherence, which unabled us to determine if one group was more adherent with their exercises than the other. Furthermore, the use of pain medication was collected at baseline but was no explored further during the course of the trial, which can be seen as a limitation. Nevertheless, not all participants were under the influence of pain medication during the trial (58% in the intervention group and 63% in the control group, shown in Table 1) making it difficult to draw any robust conclusions regarding its effect. The CT scans were not analyzed until after inclusion which resulted in a few participants being regraded from SLAC/SNAC 3 to 4. This could have affected the outcome of the trial and should be seen as a limitation. In addition, there were more men included in both the intervention and the control groups and SLAC wrist was the most common cause of OA. This is characteristic of patients with wrist OA [4], but it can limit the generalization of the current results to the whole wrist OA population.

The main strength of this trial is the assessor-blinded prospective randomized controlled design evaluating a new treatment concept for patients with wrist OA. Other strengths were that the control group received the same number of follow-ups as the intervention group with the only difference in the groups being the exercise program. However, one could argue that these exercise programs were too similar, apart from the specific exercises, making it difficult to find a difference between the groups.

### Future research and clinical implications

Since the exercise therapy program in the present trial was no better at reducing pain and improving function than a ROM training program, further research is warranted. Future studies should evaluate the most optimal type of exercise and setting for an exercise therapy program for patients with wrist OA and explore the optimal timing of a self-management treatment program regarding the grade of OA. Regarding the evaluation of pain, our RCT aimed to evaluate this subjectively with PROMs, such as the PRWE and NPRS. However, future studies should take into consideration to evaluate the existence of hyperalgesia – the presence of central sensitization–manifested as a lowered pain threshold and evaluated quantitively with an algometer, in patients with wrist OA [52]. Furthermore, to evaluate the true effects of an exercise therapy program, future studies should include, in RCTs, a control group that is not prescribed exercise as treatment or compare an exercise therapy program to different types of pharmacological or surgical treatment options.

Recruitment of patients with wrist OA for research purposes can be difficult since it is a heterogeneous condition with many different pathologies leading up to OA in one or more joints in the wrist. To increase the amount of evidence, we should therefore conduct multi-center studies in order to access a larger number of participants with wrist OA. Other future studies should also try to include a more diverse population, in order to improve the generalizability of such studies. Adherence to the exercise program is probably a key factor for the success of the treatment. Digital applications with reminders or other compliance techniques could potentially further improve adherence. We suggest that future studies of wrist OA might consider using a digital application to support adherence to a self-managed exercise therapy program.

Taken together, this is the first attempt to incorporate wrist OA in a first-line exercise therapy treatment approach. The significant and clinically important decrease in pain on load within the intervention group could have been due to the joint-protective and neuromuscular nature of the exercise therapy program. However, in this RCT, this could not be proved in a robust way. The management of patients with wrist OA in a comprehensive, multimodal first-line treatment approach, including patient education and exercises needs to be more acknowledged since it could benefit both the patient and the healthcare system.

## Data Availability

The datasets generated and/or analysed during the current study are not publicly available but available from corresponding author on reasonable request. Public access to data is restricted by the Swedish government (Public Access to Information and Secrecy Act; https://www.government.se/information-material/2009/09/public-access-to-information-and-secrecy-act/). However, data may be available for researchers upon special review and includes approval of the research project by both an Ethics Committee at national level and governmental data safety committees.

## Abbreviations

CMC: Carpometacarpal
CONSORT: Consolidated standards of reporting trials
CT: Computer tomography
DASH: Disabilities of the arm, shoulder, and hand
GROC: Global rating of change
GSES: Generalized self-efficacy scale
Kg: Kilogram
MCID: Minimal clinically important difference
NPRS: Numeric pain rating scale
NSAID: Non-steroidal anti-inflammatory drug
OA: Osteoarthritis
OT: Occupational therapist
PRWE: Patient rated wrist evaluation
PT: Physiotherapist
RCT: Randomized controlled trial
ROM: Range of motion
SD: Standard deviation
SLAC: Scaphoid lunate advanced collapse
SNAC: Scaphoid non-union advanced collapse
